# Adapting a Self-Care Intervention to Meet the Needs of Caregivers of Persons With Frontotemporal Degeneration

**DOI:** 10.1093/geroni/igaf122.018

**Published:** 2025-12-31

**Authors:** Lauren Fisher, Barbara Riegel, Karen Hirschman, Lauren Massimo

**Affiliations:** University of Pennsylvania, Philadelphia, Pennsylvania, United States; University of Pennsylvania, Philadelphia, Pennsylvania, United States; University of Pennsylvania, Philadelphia, Pennsylvania, United States; University of Pennsylvania, Philadelphia, Pennsylvania, United States

## Abstract

While many caregiver interventions focus on education about symptom management for a person with dementia, the Virtual Caregiver Coach for You (ViCCY) focuses explicitly on the caregiver’s ability to care for their own physical and emotional health (i.e., self-care). We use motivational coaching to bring awareness to caregiving demands, stressors, and coping strategies for caregivers to build support and resources. ViCCY is a virtual health coaching intervention that partners health coaches and caregivers in 1:1 relationships to enhance self-care over 10 sessions in 6 months. ViCCY was initially developed for caregivers of persons living with heart failure. Throughout development, our study aimed to adapt this intervention for Frontotemporal Dementia (FTD) based on a review of self-care literature, parent study findings in heart failure, the investigators’ clinical expertise, and focus groups to include content related to the unique symptoms of FTD and the demands in this caregiver group. The 6-month randomized controlled pilot study demonstrated significant improvement in self-care monitoring and confidence (two of three domains on the self-care inventory) for the intervention group (n = 15) compared to a control group (n = 15). We conducted focus groups after the completion of the pilot to receive feedback from participants about their experience in the intervention group. Caregivers appreciated the structured protocol content (including self-care, stress, and coping) but suggested integrating more specialized information, such as caregiver anticipatory grief. Based on the pilot study findings and focus group feedback, we have adapted the intervention protocol for the next phase of this intervention.

